# Assessment of health literacy in the adult population registered to family medicine physicians in the Republic of Srpska, Bosnia and Herzegovina

**DOI:** 10.1080/13814788.2019.1571579

**Published:** 2019-02-22

**Authors:** Nevena Todorovic, Aleksandra Jovic-Vranes, Bosiljka Djikanovic, Natasa Pilipovic-Broceta, Nadja Vasiljevic, Vesna Lucic-Samardzija, Aleksandar Peric

**Affiliations:** a Faculty of Medicine, Family Medicine Department, University of Banja Luka, Banja Luka, Bosnia and Herzegovina;; b Faculty of Medicine, Institute of Social Medicine, University of Belgrade, Belgrade, Serbia;; c Faculty of Medicine, Institute of Hygiene and Medical Ecology, University of Belgrade, Belgrade, Serbia;; d Primary Healthcare Center Banja Luka, Banja Luka, Bosnia and Herzegovina

**Keywords:** Health literacy, primary healthcare, Bosnia and Herzegovina

## Abstract

**Background:** Health literacy is an important determinant of health. This concept is under-researched in the Republic of Srpska, Bosnia and Herzegovina.

**Objectives:** To assess health literacy and its association with sociodemographic variables, self-perception of health and the presence of chronic conditions in primary healthcare setting.

**Methods:** In May 2016, a cross-sectional study was executed in two primary healthcare centres. Out of approximately 1500 patients who visited both health centres during four consecutive days, about 800 were eligible. Of these, 110 patients agreed to complete the translated Short Test of Functional Health Literacy in Adults (S-TOFHLA). The influence of demographic, social, economic, and health characteristics (independent variables) on the S-TOFHLA score (dependent variable) was assessed by multiple logistic regression analysis.

**Results:** One questionnaire was incomplete and therefore 109 questionnaires were analysed. Inadequate, marginal, and adequate health literacy were present in 19 (17.4%), 16 (14.7%) and 74 (67.9%) respondents. Adequate health literacy was found predominantly among respondents younger than 55 years and those with a high level of education. Regression analyses showed that low level of education (OR: 5.3), age 55 years and over (OR: 3.9), living in a rural area (OR: 3.7) and having three or more chronic diseases (OR: 2) were independently associated with inadequate or marginal health literacy.

**Conclusion:** In this study performed in two primary healthcare centres in the Republic of Srpska, Bosnia and Herzegovina, low health literacy was associated with low level of education, older age, living in a rural area, and having more chronic diseases.

KEY MESSAGES

- In the Republic of Srpska, Bosnia and Herzegovina, low health literacy is associated with lower level of education, age over 54 years, living in a rural area, and having three or more chronic diseases.
- This information may be useful for the work of family doctors and the provision of health services.

## Introduction

The health literacy (HL) of a population can be described as a ‘Health literacy is linked to literacy and entails people’s knowledge, motivation and competences to access, understand, appraise, and apply health information to make judgments and take decisions in everyday life concerning healthcare, disease prevention and health promotion to maintain or improve quality of life during the life course’ [1]. Information about the HL level of European populations was scarce until results of the ‘European Health Literacy Project 2009–2012’ were published [2–5]. During the past three decades, the population in the Republic of Srpska, Bosnia and Herzegovina (RS, B&H) suffered a tough period marked by war, migration, post-war transformation in political, cultural, and social spheres of life, and reforms in the healthcare system. Considering that 4.3% of the population of the RS (B&H) is illiterate and that the migration affected educational and earning opportunities, the lack of education and poor living conditions could significantly affect the morbidity and HL. Previous research showed the increased prevalence of marginal HL worldwide [2,6]. No previous studies have examined HL in RS, B&H.

Therefore, this study intended to raise the interest of health workers and institutions responsible for HL and emphasize its importance in the health-providing services, which can ultimately improve health and the welfare of citizens. This study aimed to evaluate the level of HL of primary healthcare users in two cities in the RS (B&H), and to identify and analyse factors that affect the HL level.

## Methods

### Setting and study sample

This cross-sectional study was carried out among patients registered with family physicians in two primary healthcare centres Prijedor and Bijeljina (north-western and north-eastern parts of the RS, B&H), in the period from 3–13 May 2016. The study was conducted in accordance with the World Medical Association Declaration of Helsinki (2008), after the approval from the Ethics Committees of the Faculty of Medicine Belgrade and the primary healthcare centres in Prijedor and Bijeljina. Two trained investigators-physicians conducted the survey. Each respondent received a written explanation of the goals and methods of this research and they were asked to sign the informed consent form before enrolment into this study.

### Selection and description of participants

Out of approximately 1500 patients who visited the health centres for any reason—in Prijedor during the first four days of the research period and in Bijeljina during the consecutive four days—about 800 patients met the inclusion criteria. The main inclusion criterion was the respondent’s age being over 18 years old. Respondents unwilling to cooperate, illiterates, individuals not being able to communicate in the Serbian language, those with visual disorders, terminal stage of disease, organic brain damage, mental disorder or alcohol intoxication were excluded. Individuals with a higher professional education or university diploma in medicine, dentistry or pharmacy, were excluded from the study. Finally, 110 eligible patients agreed to participate in the research (i.e., approximately every eighth respondent).

### Data collection

Short Test of Functional Health Literacy in Adults (S-TOFHLA) and a sociodemographic questionnaire were administered by investigators-physicians after the respondents received a health service (visit to a family doctor or nurse). Respondents individually gave written answers to questions from both questionnaires. It took approximately 30 min for each respondent to complete both questionnaires (application of criteria for exclusion from the study, explanation of objectives and methods of research, written consent for participation, and completion of both questionnaires).

### Instruments and procedure

We used the short version of the TOFHLA questionnaire [7]. The English version of the questionnaire was translated into a structured procedure, including forward and backward translations [8]. Two independent bilingual translators (health professionals) were involved, both native Serbian speakers and fluent in English and medical terminology. Since two versions were available, both were synthesized into a single Serbian version. A third bilingual translator performed the back to compare the back-translated version with the original one and to ensure that no loss of meaning or context occurred during the translation process. Following these processes, we did not judge it necessary to make any additional modifications of the Serbian translation or cultural adaptation. The cultural adaptation of the questionnaire was performed by recruiting 10 patients (five from each primary healthcare centre) and rated as clear and understandable. The purpose of this cultural adaptation was to provide a version understandable by patients and a version that would be conceptually identical to the original version of the questionnaire

Along with the S-TOFHLA, a sociodemographic questionnaire was developed. This questionnaire included 23 items on demographic, social, economic, as well as health characteristics. We measured self-perceived material status and health using five-point Likert scales (very bad, bad, average, good, very good), while for analysis; a three-point scale was used. The health characteristics of the respondents encompassed self-assessment of general health; use of health service; the presence of chronic illness; and bad habits. The use of health services was evaluated through the number of visits to family medicine specialists, other specialists in the state and private sector, and the number of hospitalizations over the past 12 months. Respondents were asked to list any chronic diseases and medications taken (used at least seven days due to the illness). Respondents were asked about their risk behaviour, which included smoking, alcohol intake, body mass index and insufficient physical activity. We measured self-perceived life satisfaction using five-point Likert scale (not satisfied, slightly satisfied, satisfied, quite satisfied, completely satisfied) while for analysis a three-point scale was used.

### Description of the instrument

The questionnaire was divided into two parts—A and B (which fully correspond to the passages from the standard version of the questionnaire) with different values of the Gunning Fog index. This index denotes the ease of understanding the text in the first reading, and is the result of the length (number of years) of education, thus the index for the first part is 4.3 and for the second is 10.4. If it is less than 12, it is intended for a large part of the population. Part A consists of 16 items and refers to the recognition of the instructions that the patient receives before performing diagnostic procedures for the upper gastrointestinal tract examination. Part B has 20 items, examines the knowledge of patients’ rights and obligations in the healthcare system. The time provided for completing the questionnaire is 7 min. Understanding of the written information is estimated by excluding the fifth or seventh word in each part of the text, and the respondents should choose one of the four words offered that best fits in the context of the sentence (modified Cloze method). The Cloze method demonstrates the ability of the respondent to understand the context and, therefore, to choose the right word that gives meaning to the information.

The respondents got 1 point for each correct answer, and 0 points for an incorrect answer, so the maximum number of possible points obtained in the shortened version of questionnaire is 36. Health literacy was classified as inadequate HL (score, 0–16), marginal HL (score, 17–22), and adequate HL (score, 23–36) [7].

### Statistical analysis

Data were entered and analysed using the Statistical Package for Social Sciences (SPSS), version 22 (IBM Corporation, Armonk, NY, USA). The chi-squared test was used to assess the significance of differences by patient characteristics and functional HL categories. Demographic, social, economic, and health characteristics were independent variables and HL was a dependent variable. Their relationship was assessed by multiple logistic regressions. The HL was a binary outcome high HL (adequate) and low HL (inadequate and marginal). The analysis of logistic regression was organized in two stages. The probability, *p* < 0.05, was taken as the minimum level of significance.

## Results

### Sociodemographic characteristics of respondents

The S-TOFHLA questionnaire was administered among 110 patients, one questionnaire was incomplete and excluded from the analysis; therefore, 109 questionnaires were analysed. The demographic data are shown in Table 1. Women were slightly overrepresented; the average participant age was 53 years (range: 20–83 years). Two-thirds of all participants (71; 65.1%) had completed secondary education. Most respondents lived in suburban and rural areas. Moreover, 34 (31.2%) of the respondents were in exile or displaced by war. Only 47 (43.1%) of the respondents were employed full-time. Fifty-five respondents (50.5%) reported their material status as average.

**Table 1.** S-TOFHLA score mean values by sociodemographic and medical characteristics of the respondents.

		S-TOFHLA^a^	
	*n* (%)	Mean	Standard deviation
Gender			
Male	52 (47.7)	26.3	9.2
Female	57 (52.3)	26.4	9.1
Age, years			
≤44	36 (33.0)	31.0	5.8
45–54	19 (17.4)	30.5	5.5
55–64	27 (24.8)	25.1	9.5
≥65	27 (24.8)	18.4	8.8
Marital status			
Married	83 (76.1)	26.2	9.4
Other	26 (23.9)	26.8	8.0
Education			
Primary school or less (≤8)	18 (16.5)	20.4	9.4
High school (8–12)	71 (65.1)	26.2	9.1
University	20 (18.4)	32.2	4.2
Area			
Urban	48 (44.0)	27.9	8.6
Rural	61 (56.0)	25.1	9.3
Change of residence			
Yes	34 (31.2)	25.9	8.2
No	75 (68.8)	26.5	9.5
Employment			
Employed	47 (43.1)	30.4	6.5
Other (unemployed, retired, pupil, student, housewife)	62 (56.9)	23.3	9.6
Material status			
Poor	13 (11.9)	22.0	8.7
Average	55 (50.5)	25.3	9.8
Good	41 (37.6)	29.2	7.5
Life satisfaction			
Low	11 (10.1)	20.7	9.1
Moderate	50 (45.9)	24.4	10.2
High	48 (44.0)	29.6	6.5
Family doctor visits			
No visits	22 (20.2)	28.8	6.8
1–2	24 (22.0)	30.5	5.9
3–4	14 (12.8)	28.9	8.2
5–10	20 (18.3)	22.6	9.2
>10	29 (26.6)	22.3	10.8
Total	109	26.3	9.1

^a^S-TOFHLA (score 0–36).

### Health characteristics of respondents

As many as 75 (68.8%) of the respondents reported a chronic illness. The average number of chronic illnesses among the respondents was 2.7 (SD = 1.7). The number of health visits varied between 0 (20.2%) and over 10 (26.6%) (Table 1).

### Health literacy of respondents

Inadequate marginal HL was found in 35 (32.1%) respondents. Correct answers to all the questions from the part A were given by 52 (47.7%) respondents, and only one (0.9%) respondent gave correct answers to the question in part B. Regarding the S-TOFHLA test, the mean S-TOFHLA score was 26.3. The worst results were obtained among respondents over 65 years (mean score 18.4) (Table 1). Relatively high S-TOFHLA scores above 30 were seen among patients with a university education (score 32.2), employed (score 30.4) aged 45–54 years, and those who visited their health centre once or twice a year (Table 1). Good health status and adequate HL were reported by 48 (64.8%) of the respondents (Table 2).

**Table 2.** Distribution of health literacy levels according to the sociodemographic and medical characteristics of the respondents. Data is given as number and row-percentages (%).

	STOFHLA			
Characteristics	Inadequate	Marginal	Adequate	*p*-value^a^
	*n* (%)	*n* (%)	*n* (%)	
Area				0.017
Urban (*n* = 48)	8 (16.7)	2 (4.2)	38 (79.1)	
Rural (*n* = 61)	11 (18.0)	14 (23.0)	36 (59.0)	
Gender				0.856
Male (*n* = 52)	10 (19.2)	8 (15.4)	34 (65.4)	
Female (*n* = 57)	9 (15.8)	8 (14.0)	40 (70.2)	
Age, years				0.000
≤44 (*n* = 36)	2 (5.5)	2 (5.5)	32 (88.9)	
45–54 (*n* = 19)	1 (5.3)	1 (5.3)	17 (89.4)	
55–64 (*n* = 27)	6 (22.2)	4 (14.8)	17 (63.0)	
≥65 (27)	10 (37.0)	9 (33.3)	8 (29.7)	
Marital status				0.308
Married (*n* = 83)	16 (19.3)	10 (12.0)	57 (68.7)	
Other (*n* = 26)	3 (11.5)	6 (23.1)	17 (65.4)	
Employment				0.001
Employed (*n* = 47)	3 (6.4)	3 (6.4)	41 (87.2)	
Other (unemployed, retired, pupil, student, housewife) (*n* = 62)	16 (25.8)	13 (21.0)	33 (53.2)	
Education				0.005
Primary school or less (≤8) (*n* = 18)	5 (27.8)	6 (33.3)	7 (38.9)	
High school (8–12) (*n* = 71)	14 (19.7)	9 (12.7)	48 (67.6)	
University (*n* = 20)	0 (0.0)	1 (5.0)	19 (95.0)	
Material status				0.154
Poor (*n* = 13)	4 (30.8)	3 (23.0)	6 (46.2)	
Average (*n* = 55)	12 (21.8)	7 (12.7)	36 (65.5)	
Good (*n* = 41)	3 (7.3)	6 (14.7)	32 (78.0)	
Self-perception of health				0.007
Poor (*n* = 16)	7 (43.7)	2 (12.5)	7 (43.7)	
Average (*n* = 33)	7 (21.2)	7 (21.2)	19 (57.6)	
Good (*n* = 60)	5 (8.3)	7 (11.7)	48 (80.0)	
Chronic illness				0.000
None (*n* = 34)	0 (0.0)	3 (8.8)	31 (91.2)	
One (*n* = 26)	4 (15.4)	1 (3.8)	21 (80.8)	
Two (*n* = 17)	4 (28.6)	3 (21.4)	7 (50.0)	
Three (*n* = 10)	5 (50.0)	1 (10.0)	4 (40.0)	
More than three (*n* = 25)	6 (24.0)	8 (32.0)	11 (44.0)	
Life satisfaction				0.028
Low (*n* = 11)	4 (36.4)	2 (18.2)	5 (45.4)	
Moderate (*n* = 40)	12 (24.0)	9 (18.0)	29 (58.0)	
High (*n* = 48)	3 (6.3)	5 (10.4)	40 (83.3)	

^a^According to chi-squared test.

### Analysis of predictors of inadequate health literacy

The multiple logistic regression analysis showed a significant association between being inadequately health literate and aged over 54 years (OR: 3.88, *P* = 0.010), low level of education (OR: 5.34, *P* = 0.026), residence in a rural area (OR: 2.68, *P* = 0.032), and three or more chronic diseases (OR: 1.95, *P* = 0.016), respectively (Table 3).

**Table 3.** Factors associated with health literacy according to the STOFHLA score: adequate (1)^a^ versus marginal and inadequate (0),^b^ ORs, and 95%CIs.

	Univariate			Multiple		
Variable	OR	95%CI	*p*	OR	95%CI	*p*
Age groups						
≤44.45–54 (1), 55–64, ≥65	4.67	2.36–9.24	0.000	3.88	1.38–10.90	0.010
Area						
Rural, urban (1))	2.64	1.11–6.26	0.028	3.68	1.12–12.11	0.032
Level of education						
Primary school or less, high school, university (1)	1.04	0.45–2.42	0.001	5.34	1.22–23.46	0.026
Employment						
Retired	6.80	2.70–17.12	0.000			
Other, pupil, student, employed, unemployed, housewife (1)	1.00					
Material status						
Poor	2.83	0.87–9.18	0.083			
Average or good (1)	1.00					
Family doctor visit						
No visits, 1–2, 3–4 (1), 5–10, >10	1.57	1.17–2.11	0.003			
Chronic illness						
None, one, two (1), three or more	1.89	1.40–2.53	0.000	1.95	1.13–3.37	0.016
Self-perception of health						
Poor, average or good (1)	1.94	1.23–3.05	0.004			
Life satisfaction						
High (1)	0.44	0.26–0.75	0.003			
Moderate or low	1.00					

^a^High HL.

^b^Low HL.

OR, odds ratio; 95%CI, 95% confidence interval.

## Discussion

### Main findings

The results of our study showed that the average S-TOFHLA score for all respondents was 26.3 (SD = 9.1), and that 32% of respondents had no adequate HL. Less than half of the respondents provided all correct answers associated with the instructions on diagnostic procedures for the upper gastrointestinal tract examination. Questions related to medical information, rights and one respondent correctly answered responsibilities in the healthcare system. Multiple logistic regression analysis showed that people aged over 54 years, with a lower level of education, residence in a rural area, and three or more chronic diseases have a higher probability of being inadequately health literate.

### Comparison with other studies

Our results are similar to those in the region, as well as throughout Europe [2–4]. In the Republic of Croatia every third respondent was either inadequately or marginally health literate [4]. In the Republic of Serbia, as well as in eight countries of the European region, on average every second respondent was inadequately or marginally health literate [2,3].

There were no significant differences in gender, place of residence, marital and material status, and a number of visits to a physician while analysing the overall level of HL in our study, unlike other scientific investigations that indicated gender as a key segment of HL [3,9–12]. Moreover, results in this study indicate that low HL is present amongst the more vulnerable population groups, most notably senior people, with low educational attainment, and a higher number of chronic illnesses. Most studies have recognized that age of a respondent plays a vital role in the level of HL [2,3,9–16]. Respondents over 65 years obtained the poorest S-TOFHLA test results in our investigation, which is in line with a US study [9]. The best S-TOFHLA test results were obtained from the respondents with a high level of education, showcasing education as a significant segment of HL, as demonstrated by the results of other studies [3,9,11–17]. Our results also highlighted that employed respondents had a higher level of HL, as reported by other investigators [2,9–11,15]. In addition, the HL of a respondent significantly depends on health status as well as a higher number of chronic illnesses [2,3,9,12,15,18,19].

Multivariate logistic regression in this study shows that age, level of education, place of residence and three or more chronic illnesses are relevant and statistically significant associated with HL level. In other studies gender, employment, material and marital status, self-assessment of health and life satisfaction are significant [3,15,18,20–22].

### Implications

Family doctors in RS (B&H) often encounter the inadequate HL of their patients, having problems with the demanding implementation of health promotion and preventive activities, problematic use of clinical guidelines in diagnostics and treatment and reduced compliance to medication.

This paper presents the first results of the assessment of HL and its association with sociodemographic variables, self-assessment of health, the presence of chronic diseases and the material status of patients in primary healthcare in the territory of the RS (B&H). Results show that primary healthcare providers in RS (B & H) need to adapt health information to patients’ age, level of education, place of residence and the number of their illnesses. In the forthcoming period, it is necessary to examine the level of HL on a larger sample of our population and to introduce the obtained results to policymakers with the aim of planning interventional activities to raise the level of HL.

### Study limitations

This study was conducted in the two cities in the RS (B&H), so the findings cannot be generalized to the whole RS. The study sample size is small and our results need to be interpreted with caution. The limitation of this study may also be the potential bias of patient selection. We assume that low HL level could be a reason for a refusal to participate in the study. Furthermore, despite the strong recommendations and detailed information provided by the authors of the study, we cannot rule out the choice of patients based on the preferences of investigators-physicians.

## Conclusion

Among patients visiting primary care centres in RS (B&H), low health literacy was found in almost one-third of the respondents. Marginal and inadequate health literacy was associated with a lower level of education, aged older than 54 years, not living in urban areas, and a higher number of chronic illnesses.
